# Chloroplast Haplotype Analysis Reveals High Genetic Similarity Among Central Asian *Prunus* Species

**DOI:** 10.3390/ijms27177566

**Published:** 2026-08-24

**Authors:** Ulzhan Manapkanova, Nazgul Rymkhanova, Stefanie Reim, Eric Fritzsche, Henryk Flachowsky, Svetlana V. Kushnarenko

**Affiliations:** 1 Institute of Plant Biology and Biotechnology, 45 Timiryazev Str., 050040 Almaty, Kazakhstan; n.rymkhanova@gmail.com; 2 Faculty of Biology and Biotechnology, Al-Farabi Kazakh National University, 71 Al-Farabi Av., 050040 Almaty, Kazakhstan; 3 Julius Kühn-Institute (JKI), Federal Research Centre for Cultivated Plants, Institute for Breeding Research on Fruit Crops, 3a Pillnitzer Platz, 01326 Dresden, Germany; stefanie.reim@julius-kuehn.de (S.R.); eric.fritzsche@julius-kuehn.de (E.F.); henryk.flachowsky@julius-kuehn.de (H.F.)

**Keywords:** *Prunus* spp., wild cherry species, chloroplast regions, restriction endonucleases

## Abstract

Genetic variation in four wild *Prunus* taxa (*P. fruticosa*, *P. erythrocarpa*, *P. verrucosa* and *P. griffithii* var. *tianshanica*) was investigated for the first time using six chloroplast DNA regions (*matK*, r *rpl16*, *ycf1_1*, *ycf1_2*, *ndhF* and *trnH–psbA*) analysed through CAPS-based SNP detection. The results revealed weak chloroplast differentiation among *P. erythrocarpa*, *P. verrucosa* and *P. griffithii* var. *tianshanica.* However, chloroplast variation exhibited a strong geographic signal across the studied populations. The observed chloroplast variation primarily reflected geographic structuring rather than clear differentiation among these closely related taxa. In contrast, *P. fruticosa* showed distinct chloroplast haplotypes not shared with the other taxa. These findings demonstrate that the developed chloroplast CAPS marker system is effective for detecting chloroplast haplotype variation but has limited discriminatory power among closely related wild *Prunus* taxa. Further studies using nuclear markers and genome-wide approaches will be required to better resolve their genetic relationships and evolutionary history.

## 1. Introduction

The genus *Prunus* (subfamily *Prunoideae* of the family *Rosaceae*) includes approximately 400 species of trees and shrubs, many of which are of high economic value [1]. The taxonomy of the genus *Prunus* L. has long been a subject of debate. According to the classical morphological system proposed by Rehder (1940) [2], the genus was divided into five subgenera: *Prunus*, *Amygdalus*, *Cerasus*, *Padus*, and *Laurocerasus*. Within this framework, subgenus *Cerasus* encompassed a broad range of cherry species. Subsequently, Ingram (1948) [3] proposed a further subdivision of this group, separating related taxa into the distinct groups *Cerasus* and *Lithocerasus*. In later taxonomic treatments, Rehder’s classification continued to serve as the foundation of morphological systematics within the genus.

However, with the advent of molecular approaches, interpretations of intrageneric relationships have changed substantially. Phylogenetic studies based on chloroplast genome data, including the work of Shen et al. (2023) [4], as well as earlier molecular analyses using ITS and chloroplast *trnL–trnF* sequences [5], have supported a more consolidated classification scheme. According to this modern framework, the genus *Prunus* is divided into three principal subgenera: *Padus*, *Prunus*, and *Cerasus*.

Central Asia, including the territories of present-day Kazakhstan and Uzbekistan, is considered one of the key regions for the origin and conservation of biodiversity within the genus *Prunus*. According to phylogenetic and biogeographic reconstructions, approximately 51–53 million years ago, Western China and Central Asia—particularly the Himalayan region—were likely centers of origin and early diversification of the subgenus *Cerasus*. During the Miocene, Central Asia played a crucial role in the westward migration of *Prunus* representatives, serving as a biogeographic corridor between East Asia and Europe following the closure of the Turgai Strait. The complex geological history of the region, including the uplift of the Qinghai–Tibetan Plateau and subsequent climatic fluctuations, facilitated both population isolation and the formation of local lineages, resulting in high diversity and endemism of wild cherry species. Despite this, the genetic structure and evolutionary relationships of many Central Asian *Prunus* taxa remain insufficiently studied to date [4,6]. *Prunus verrucosa* was first described by Adrien René Franchet in 1883 [7] and represents one of the earliest formally recognized wild cherry taxa of Central Asia. Later, in 1948 [3], Ingram revised the taxonomic status of *P. griffithii* var. *tianshanica* (Pojark.) Ingram, and in 1966 [8], Gilli established *P. erythrocarpa* (Nevski) Gilli as an independent species [9,10,11]. Despite the historical documentation of these taxa, available information regarding their molecular genetic structure, intraspecific diversity, and evolutionary relationships remains limited.

Traditionally, cherry species are identified in the field based on morphological traits such as leaves, inflorescences, flowers, and fruits. However, the literature indicates that, due to substantial overlap in species distributions, it is very difficult to distinguish interspecific differences from intraspecific variation, and the delimitation of species boundaries within this subgenus remains a controversial issue. Even the subdivision within the subgenus based on morphological characteristics remains controversial and requires validation using complementary sources, including molecular data [4,12]. The presence of a large number of species within the genus *Prunus* increases the potential for interspecific hybridization and significantly complicates the establishment of a clear botanical classification.

Previously, we investigated the genetic diversity of four cherry species indigenous to Kazakhstan and Uzbekistan using SSR markers. These markers allowed us to distinguish *Prunus fruticosa* from three other species—*P. griffithii* var. *tianshanica*, *P. erythrocarpa*, and *P. verrucosa*; however, clear differentiation among these three co-occurring species remained challenging [13]. This is particularly evident when compared to the more extensively studied *Prunus* species of East Asia and Europe, where phylogenetic analyses and population genetic analyses using nuclear and organellar markers have substantially clarified their evolutionary history and interspecific relationships [4,14].

There are numerous examples of the use of molecular genetic approaches to elucidate genetic relationships, levels of divergence, and the evolutionary history of closely related wild species based on nuclear (SSR) and chloroplast markers [14,15,16]. Chloroplast markers are widely used in phylogeographic and evolutionary studies due to their conservative inheritance, predominantly maternal transmission, and relatively low mutation rate [17,18]. The CAPS (Cleaved Amplified Polymorphic Sequences) approach allows cost-efficient screening of chloroplast variation across multiple individuals [19] and has been successfully used to study the phylogenetic relationships of cultivated species of the genus *Panax* [20] and *Prunus* [1].

The aim of the present study was to characterize chloroplast haplotype variation and the genetic structure of populations in four wild *Prunus* taxa from Kazakhstan and Uzbekistan using six chloroplast CAPS markers, as well as to assess the informative value and limitations of the developed markers for identifying chloroplast variability in closely related taxa.

## 2. Results

### 2.1. Haplotype Analysis

Using the HAPLOTYPE ANALYSIS software (version 1.05), 43 haplotypes were identified in the 121 samples analyzed, demonstrating the presence of considerable chloroplast genetic variation within the studied material. Most haplotypes occurred at low frequencies, indicating substantial within-species variation. Table 1 summarizes the genetic diversity parameters for the investigated populations. The observed genetic diversity varied among populations, with the number of haplotypes ranging from 1 to 13 and gene diversity (He) from 0.000 to 1.000 (mean = 0.729). Several populations contained multiple private haplotypes, whereas others showed no private variation.

**Figure 1:**
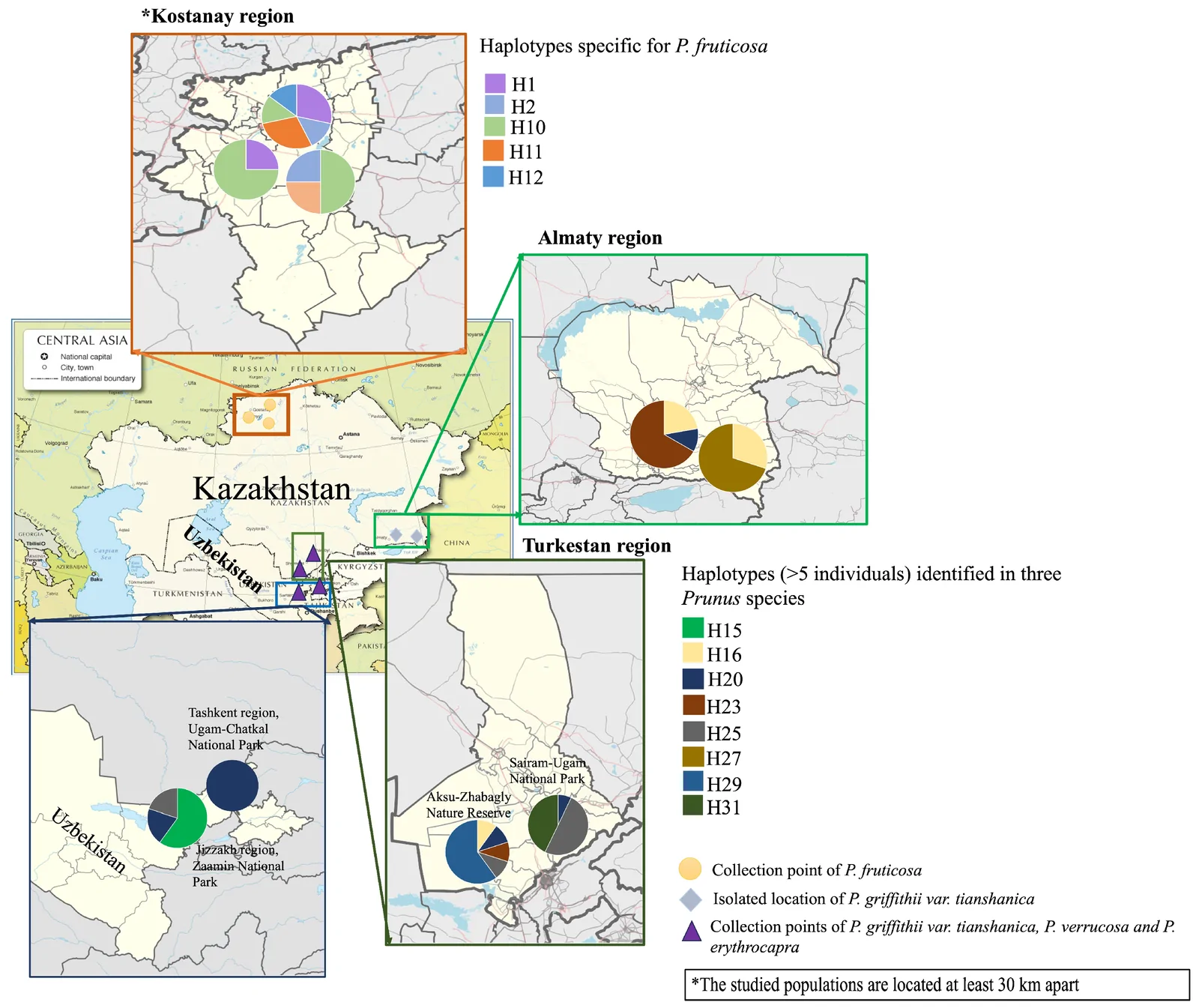
Geographic distribution of chloroplast haplotypes identified among wild *Prunus* species from Kazakhstan and Uzbekistan. Different colors represent distinct chloroplast haplotypes. Symbols indicate the geographic locations where plant materials were collected. Yellow circles correspond to *Prunus fruticosa*, blue diamonds to *P. griffithii* var. *tianshanica*, while purple triangles indicate sampling sites where three *Prunus* species were collected. The colored squares represent the sampling regions, and each square color corresponds to the haplotype composition displayed in the associated pie charts. The map illustrates the spatial distribution of chloroplast haplotypes across the studied populations.

No distinct species-specific chloroplast haplotypes were detected among the accessions of *Prunus erythrocarpa*, *P. griffithii* var. *tianshanica*, and *P. verrucosa* from Kazakhstan and Uzbekistan. Instead, haplotypes were extensively shared among the three taxa, and their distribution did not correspond to the current taxonomic classification. The most frequent haplotypes (H15, H16, H20, H23, H25, H27, H29 and H31) occurred across multiple taxa (Figure 1), indicating that chloroplast variation does not support species-level differentiation within this group.

In contrast, some haplotypes showed restricted geographic distributions. Haplotype H27 was detected exclusively in seven accessions of *P. griffithii* var. *tianshanica* from the Big Aksu population (Almaty region, Kazakhstan), whereas haplotype H29 was found only in six accessions of *P. verrucosa* from the Aksu-Zhabagly Nature Reserve (Turkestan region, Kazakhstan) (Figure 1). These patterns indicate population-specific geographic structuring of chloroplast variation rather than taxon-specific differentiation.

Five haplotypes (H1, H2, H10, H11 and H12) were identified in *P. fruticosa* (Figure 1). In contrast to the other *Prunus* species analyzed, these haplotypes were exclusively found in *P. fruticosa*, with no sharing observed among the three other species from Kazakhstan and Uzbekistan.

### 2.2. Barcoding Potential

**Figure 2:**
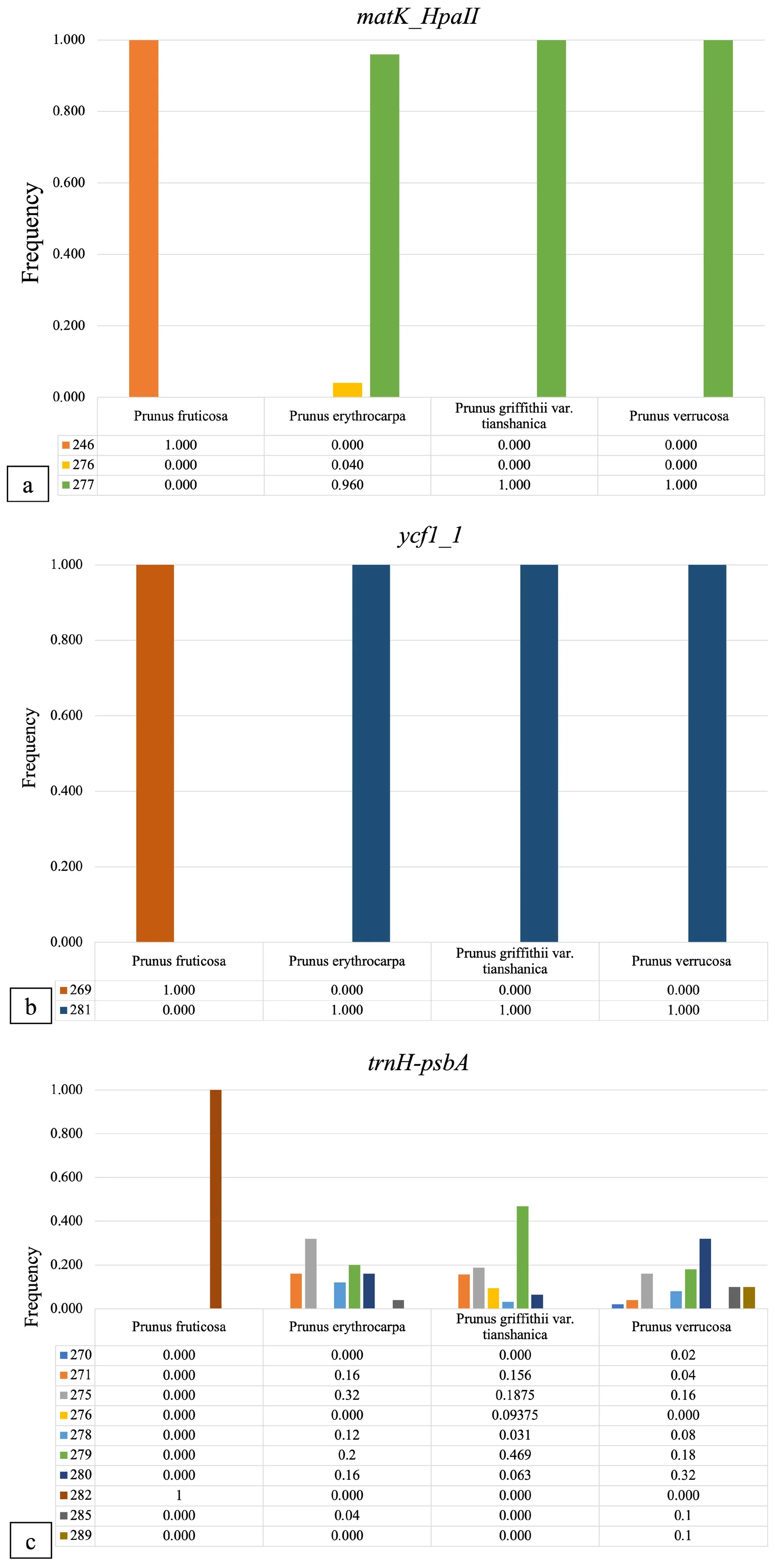
Identified alleles in *matK_HpaII* (**a**), *ycf1_1* (**b**) and *trnH-psbA* (**c**) regions for four species of *Prunus.* Bars represent the frequency of each allele, and the accompanying table beneath the histograms summarizes the distribution of alleles among the analyzed species.

All results indicate that there is no clear differentiation based on chloroplast markers among the three species, *P. griffithii* var. *tianshanica*, *P. erythrocarpa* and *P. verrucosa*. At the same time, *P. fruticosa* demonstrated genetic distinctiveness. For the three studied populations of *P. fruticosa*, 100% private alleles were identified using the chloroplast markers *matK_HpaII*, *ycf1_1* and *trnH-psbA* (Figure 2). These findings suggest that these markers may be useful for the identification of *P. fruticosa* within the populations analyzed in this study. However, additional sampling across a broader geographic range is required to determine whether these haplotypes are representative of the species as a whole.

During the analysis of the data, subtle differences in the lengths of the fragments were also observed. In *P. erythrocarpa*, a 276 bp allele was identified in the *matK_HpaII* region in one accession, whereas the remaining samples of this species exhibited a 277 bp allele (Figure 2a). In the *ycf1_2* region, following restriction with the *HaeIII* enzyme, a 243 bp fragment was detected in 7 out of 13 accessions collected in Uzbekistan, whereas in the remaining accessions, a 182 bp fragment was detected (Table S1). Notably, a 243 bp fragment was also found in one accession of *P. verrucosa* (Prun24_122) collected in the Turkestan region (Kazakhstan). The highest level of fragment variability was observed in the *trnH–psbA* chloroplast region (Figure 2c).

The initial fragment of the chloroplast region *ndhF* was 696 bp in all four *Prunus* species. Digestion with the restriction enzyme *AluI* resulted in two fragments of approximately 280 and 240 bp in the majority of accessions. However, in 34 samples representing three Central Asian taxa (9 samples of *P. erythrocarpa*, 18 samples of *P. verrucosa* and 7 samples of *P. griffithii* var. *tianshanica*), an alternative restriction profile was observed, consisting of three fragments measuring 240, 190 and 90 bp. It was noted that this restriction profile was predominantly detected in samples collected in Kazakhstan, with the exception of a single *P. verrucosa* sample (Prun24_149) originating from the IV population of the Zaamin National Park in the Jizzakh Region of Uzbekistan.

In the studied chloroplast region *ycf1_1* (Figure 2b), a 269 bp initial fragment was detected in all *P. fruticosa* accessions, whereas the corresponding fragment in the other three studied species was 281 bp in length. However, following restriction of the region with the *AluI* enzyme, a fragment 143 bp in length was detected in one *P. fruticosa* sample, in contrast to the typical 106 bp fragment characteristic of other samples of this species. Additionally, two allelic fragments of 323 and 346 bp in length were identified in the *rpl16* region of *P. fruticosa*, which were absent from the other species studied. Furthermore, the 346 bp fragment was detected only in accessions from the first and second populations (Table S1).

The *Prunus* accessions in the gene bank of the Julius Kühn Institute (JKI) in Dresden-Pillnitz were also examined for the presence of private alleles. The results revealed alleles that were exclusively detected within the analyzed accessions, as well as rare alleles with limited distribution. Some *Prunus* accessions representing *P. laurocerasus*, *P. maackii*, *P. maximowiczii*, *P. padus* and *P. tomentosa* are characterized by the presence of private alleles with a frequency of 1.0 (Table 2).

The number of samples analysed for each species was as follows: two samples of *P. laurocerasus*, one sample each of *P. maackii* and *P. maximowiczii*, one sample of *P. padus* and two samples of *P. tomentosa*. Due to the limited number of accessions available for some species, these alleles should be considered putative private alleles rather than definitive species-specific markers.

### 2.3. Nei’s Genetic Identity

The Pairwise Population Matrix of Nei’s Genetic Identity based on chloroplast markers confirmed the previous analysis. Based on the developed chloroplast CAPS markers, the three species native to Kazakhstan and Uzbekistan demonstrated a high degree of genetic similarity (approximately 97–99%), while *P. fruticosa* showed a distinct position in the genetic identity analysis (Table 3).

However, these estimates reflect similarity only within the analyzed chloroplast regions and maternal lineages and should not be interpreted as evidence of genome-wide genetic relationships or overall species similarity.

### 2.4. Phylogenetic Relationships and Geographical Structure of the Four Prunus Species from Kazakhstan and Uzbekistan in Comparison with Other Prunus Taxa

**Figure 3:**
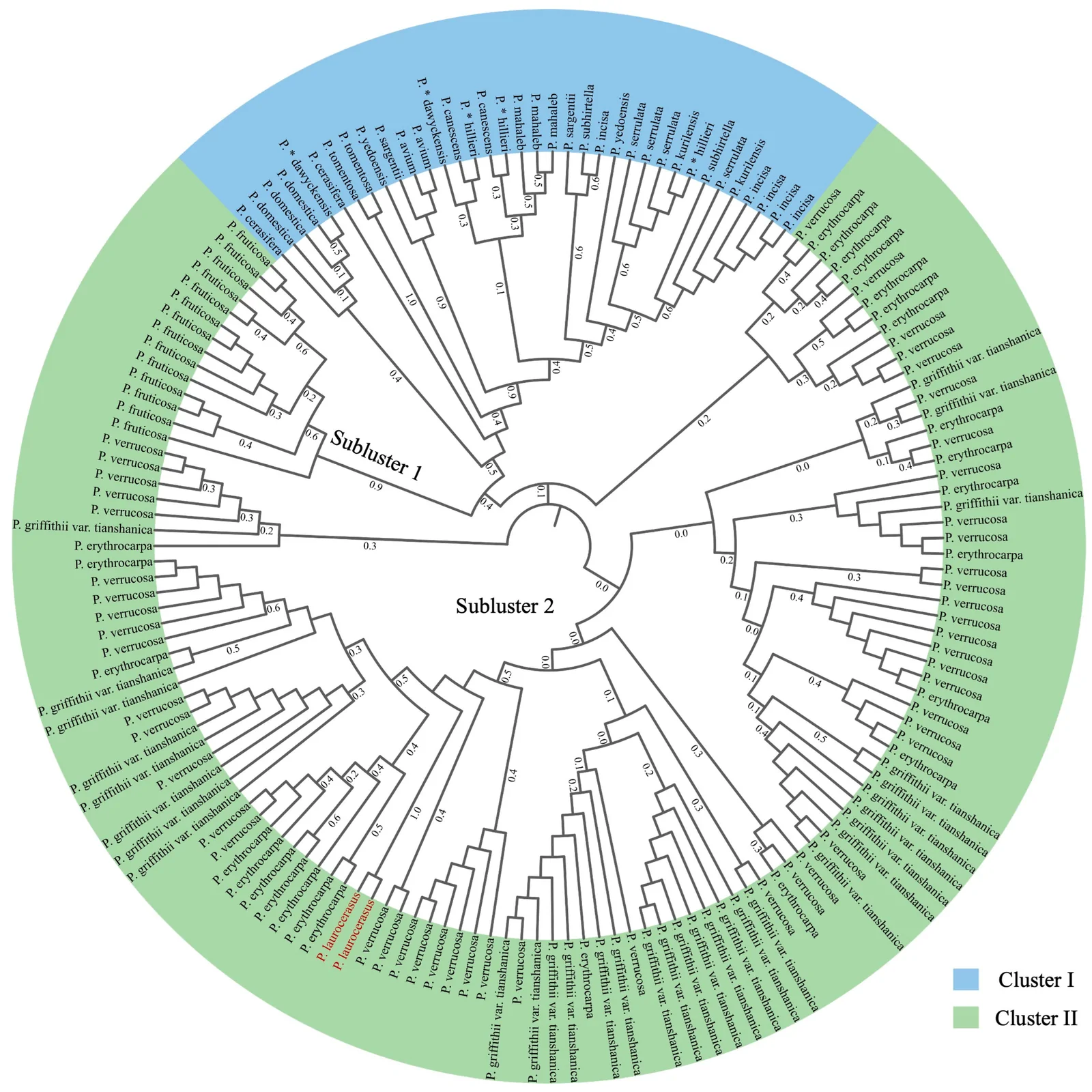
Neighbor-Joining phylogenetic tree based on chloroplast marker allele dissimilarity, reconstructed using 5000 bootstrap replicates. The tree includes 121 wild *Prunus* accessions from Kazakhstan and Uzbekistan together with 38 reference accessions from the Julius Kühn Institute (JKI) genebank (Germany). Cluster I and Cluster II are highlighted in different colors. *Prunus laurocerasus* reference accessions from the JKI collection are indicated in red font.

The phylogenetic tree was constructed using 121 accessions of four *Prunus* species from Kazakhstan and Uzbekistan and 38 accessions of 15 wild *Prunus* species from the Fruit GenBank of the Institute of Breeding Research on Fruit Crops of the Julius Kühn Institute (JKI) in Dresden-Pillnitz. Two main clusters were identified based on an analysis of chloroplast regions (Figure 3). Cluster I comprised primarily accessions of *Prunus* species from the Fruit Genebank of the Institute of Breeding Research on Fruit Crops of the Julius Kühn Institute (JKI) in Dresden-Pillnitz, whereas the Central Asian accessions (Kazakhstan and Uzbekistan) were grouped within cluster II.

Cluster I contains 36 accessions (14 species: *P. cerasifera*, *P. domestica*, *P.* × *dawyckensis*, *P. tomentosa*, *P. yedoensis*, *P. sargentii*, *P. avium*, *P. canescens*, *P. mahaleb*, *P.* × *hillieri*, *P. subhirtella*, *P. incisa*, *P. serrulata*, and *P. kurilensis*) from the JKI collection.

Cluster II consists of two subclusters. Subcluster 1 contains all 14 accessions of *P. fruticosa*. Subcluster 2 includes all Kazakhstan and Uzbekistan accessions of three studied species: *P. erythrocarpa*, *P. verrucosa* and *P. griffithii* var. *tianshanica*. However, two accessions of *P. laurocerasus* were placed within this cluster.

Overall, the chloroplast phylogeny revealed incomplete separation among the three studied *Prunus* species of Kazakhstan and Uzbekistan (*P. erythrocarpa*, *P. verrucosa* and *P. griffithii* var. *tianshanica*).

### 2.5. Geographical Structure

**Figure 4:**
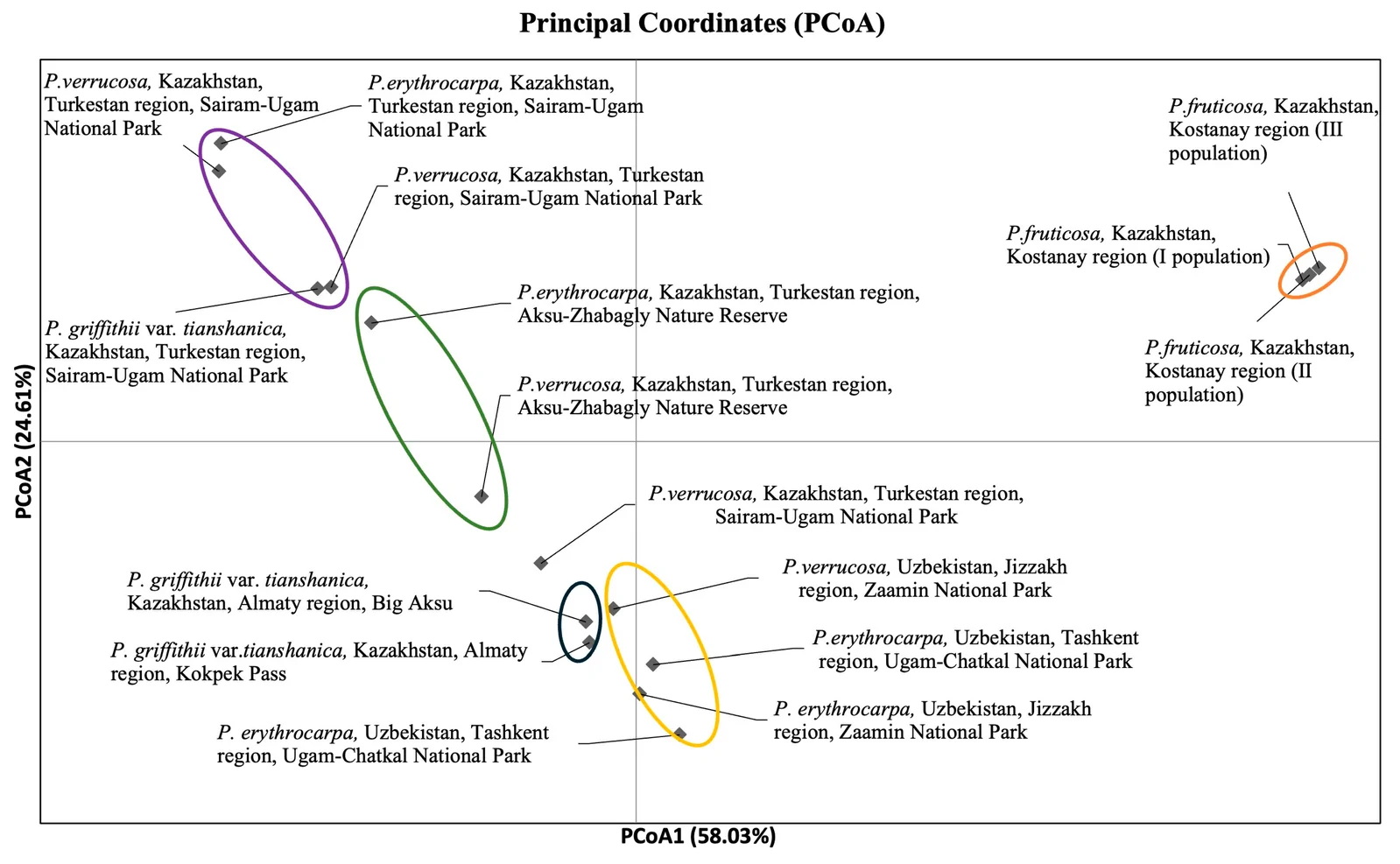
Principal coordinate analysis (PCoA) performed on 121 accessions of four *Prunus* species.

Principal coordinate analysis (PCoA) revealed geographic structuring rather than taxonomic differentiation, which may be associated with the different geographic origins of the sampled accessions. The Central Asian accessions from Kazakhstan and Uzbekistan demonstrated partial overlap between species (Figure 4). Populations originating from the same or adjacent geographic regions tended to cluster more closely, even when representing different species. For example, accessions of *P. verrucosa*, *P. erythrocarpa*, and *P. griffithii* var. *tianshanica* collected in Kazakhstan, including the Turkestan region, Sairam-Ugam National Park and adjacent areas, showed close spatial clustering in the ordination diagram. Similarly, accessions of *P. verrucosa* and *P. erythrocarpa* collected from Uzbekistan formed overlapping clusters despite taxonomic differences. In contrast, three populations of *P. fruticosa* formed a clearly distinct cluster separated from the other studied species. The pronounced spatial separation of *P. fruticosa* accessions indicates a higher level of genetic differentiation and suggests a certain degree of genetic isolation of this species within the analyzed dataset.

To investigate the partitioning of genetic variation among the studied wild cherry taxa, AMOVA analyses were conducted at two hierarchical levels: first, to evaluate genetic differentiation among species, and second, to assess population-level genetic structure within each species. The AMOVA across the four wild cherry species revealed that 64% of the total molecular variation was attributable to intraspecific variation, whereas 36% was explained by differences among species. At the population level, separate AMOVA analyses conducted for each species showed variation in the degree of population structuring. *P. griffithii* var. *tianshanica* exhibited the highest proportion of genetic differentiation among populations (53%), whereas *P. fruticosa* showed the lowest level of among-population variation (18%), indicating comparatively weaker population structuring in this species (Table 4).

The ϕ_PT_ value, which is an AMOVA-based analogue of Fst, indicates a significant, moderately high level of genetic differentiation among species (ϕ_PT_ value = 0.356, *p* = 0.001). Analysis of each species’ populations individually revealed that the highest ϕ_PT_ value was found in *P. griffithii* var. *tianshanica* (0.534), followed by *P. erythrocarpa*, *P. verrucosa* and *P. fruticosa*, with ϕ_PT_ values of 0.411, 0.210 and 0.182, respectively. 

The estimated level of gene flow, expressed as Nm (haploid) = 0.904, is considered moderate to high. Gene flow between *P. fruticosa* and the other species was low (Nm < 0.3), again indicating a high level of genetic isolation in this species. In contrast, gene flow among *P. erythrocarpa*, *P. griffithii* var. *tianshanica*, and *P. verrucosa* was high, with Nm values ranging from 13.5 to 63.

At the population level, gene flow patterns varied among species, but in most cases the highest levels of genetic exchange were observed within geographically close populations. For *P. griffithii* var. *tianshanica*, the highest gene flow was detected between the populations of Big Aksu and Kokpek Pass (Nm = 4.2), where only the *P. griffithii* var. *tianshanica* species is represented. Similarly, *P. verrucosa* showed elevated gene flow within the Turkestan region, particularly between accessions from the Aksu-Zhabagly Nature Reserve and the Sairam-Ugam National Park (Nm = 11.1). Evidence of genetic exchange was also observed between populations from the Aksu-Zhabagly Nature Reserve (Kazakhstan) and the Zaamin National Park (Uzbekistan). It should be noted that, as chloroplast markers were used, these estimates primarily reflect seed-mediated gene flow.

Analyses based on Mantel’s test, using pairwise genetic and geographic distance matrices, revealed an overall significant positive correlation between genetic and geographic distances (r = 0.433, *p* < 0.001), indicating a moderate pattern of isolation by distance among the studied samples. However, the strength of this relationship differed considerably among the investigated *Prunus* species. The strongest positive correlation was observed in *P. griffithii* var. *tianshanica* (r = 0.75, *p* < 0.001), suggesting a pronounced effect of geographic separation on genetic differentiation in this taxon. In contrast, *P. fruticosa* showed a moderate positive correlation (r = 0.37, *p* = 0.004), while *P. verrucosa* exhibited only a weak but significant relationship (r = 0.217, *p* < 0.001). For *P. erythrocarpa*, no significant association between genetic and geographic distances was detected (r = −0.017, *p* = 0.5), indicating an absence of detectable isolation-by-distance patterns in this species.

## 3. Discussion

### 3.1. High Chloroplast Haplotype Diversity Among Closely Related Prunus Taxa

Despite extensive research on chloroplast genomes, distinguishing between closely related taxa within the genus Prunus remains challenging [14,21]. In the present study, the selected chloroplast markers revealed substantial haplotypic variation, with 43 haplotypes detected across the investigated samples. Overall, chloroplast variation in the studied *Prunus* species showed low genetic differentiation. However, these markers exhibited limited discriminatory power among *P. erythrocarpa*, *P. griffithii* var. *tianshanica*, and *P. verrucosa*, as several haplotypes were shared among these taxa. Similar patterns of unclear differentiation have also been reported using SSR markers and morphological traits [13]. However, these approaches characterize different components of genetic and phenotypic variation. Nevertheless, the present CAPS markers demonstrated limited discriminatory power among these closely related taxa.

A plausible explanation for this pattern is that the three taxa occur in sympatry in several locations, which may have influenced their evolutionary history through processes such as shared ancestral variation, incomplete lineage sorting, or historical gene exchange. This, in combination with the generally low resolution of chloroplast markers at the species level, may contribute to extensive haplotype sharing and a limited ability to clearly discriminate among closely related taxa.

These findings are consistent with previous studies in the genus *Prunus*, where chloroplast haplotype sharing across species is frequently reported. Such patterns have been attributed to multiple evolutionary processes, including incomplete lineage sorting, retention of ancestral polymorphisms, limited lineage divergence, and, in some cases, interspecific gene flow, including hybridization and introgression. For example, wild and cultivated populations of *P. armeniaca* have been shown to exhibit low genetic differentiation and extensive haplotype sharing across populations and related taxa [22]. Similarly, studies on flowering cherries have demonstrated that closely related *Prunus* species often share chloroplast haplotypes, possibly reflecting complex evolutionary histories involving incomplete lineage sorting and, in some cases, historical hybridization [23,24]. Furthermore, Bao et al. (2024) [25] showed that chloroplast genetic diversity in *P. mira* is closely associated with geographic distribution and habitat conditions, highlighting the combined influence of ecological and historical processes on chloroplast variation.

In contrast, *P. fruticosa* exhibited a distinct haplotype profile compared with the other three *Prunus* species. The distinct haplotype profile observed in *P. fruticosa* indicates stronger chloroplast differentiation from the other taxa investigated in this study. This pattern may reflect a different evolutionary history of *P. fruticosa* compared with the other investigated taxa; however, additional nuclear and ecological data are required to evaluate the mechanisms underlying this differentiation.

This indicates that high chloroplast haplotype diversity does not necessarily translate into species-specific differentiation. Comparable patterns of extensive haplotype sharing have been reported in other *Prunus* species [22,23,24]. Further resolution of the relationships among these closely related taxa will require nuclear markers or genome-wide sequencing approaches.

Our results reveal contrasting patterns of chloroplast differentiation within the genus: some closely related taxa share haplotypes extensively, while others form more distinct chloroplast lineages. This could be due to several factors acting together, such as incomplete lineage sorting, retention of ancestral polymorphisms, short divergence times between recently separated taxa, or past hybridization events. Chloroplast markers alone, however, cannot distinguish between these processes [4,23,26]. Analyzing the genetic structure of wild *Prunus* species is further complicated by the frequent sympatric occurrence of closely related taxa, which likely contributes to the complex and overlapping chloroplast haplotype distributions observed among *Prunus* species in natural habitats, consistent with previous findings by Baek et al. (2018) [26].

### 3.2. Geographic Structure in Central Asian Prunus

The observed geographic patterns of chloroplast haplotypes in the present study are broadly consistent with previous findings in *Prunus* [27]. A significant association between haplotype and geographic origin was detected for *P. griffithii* var. *tianshanica*, *P. fruticosa*, and *P. verrucosa*, whereas no such relationship was observed for *P. erythrocarpa*.

A clear geographic–genetic association was found in *P. griffithii* var. *tianshanica*, which showed a strong correlation between genetic and geographic distances, indicating spatial structuring among populations. The occurrence of rare and locally restricted haplotypes (e.g., H27 and H29) suggests that geographic and demographic processes, such as local drift or restricted dispersal, may have contributed to the observed pattern. Similar patterns have been reported in *Prunus spinosa* [28].

In contrast, *P. erythrocarpa* showed no detectable isolation-by-distance pattern, despite a relatively high level of genetic variation. Such decoupling between genetic and geographic structure is consistent with other woody *Prunus* taxa, including *P. spinosa* and *P. armeniaca*, where within-population variation often exceeds among-population differentiation [28,29].

Because chloroplast DNA is maternally inherited, it can retain signals of historical population processes, including migration and range changes [30,31]. Accordingly, haplotype distributions in *Prunus* (e.g., *P. mira*, *P. tomentosa*) often reflect both geographic barriers and environmental gradients [25,27]. Overall, the results suggest that chloroplast variation in *Prunus* is influenced by a combination of spatial structure, historical demographic processes, and species-specific evolutionary histories rather than by isolation by distance alone.

### 3.3. Phylogenetic Analysis Reveals Regional Patterns of Chloroplast Variation Rather than Strict Species Boundaries

The phylogenetic tree shows that all samples from Central Asia, represented by Kazakhstan and Uzbekistan, form a distinct cluster that is clearly separated from the majority of reference samples. This suggests the presence of a geographically structured chloroplast haplotype group shared among the Central Asian samples. All samples from JKI are located within the same cluster (cluster I), despite their wide taxonomic diversity. This pattern indicates that several *Prunus* species possess similar or closely related chloroplast haplotypes, suggesting limited resolution of chloroplast markers at the interspecific level. Only *Prunus laurocerasus* is placed outside this cluster, which once again highlights its distinct phylogenetic position.

The inclusion of *P. laurocerasus* in the Central Asian cluster may reflect the limited resolution of the chloroplast markers used or the presence of shared ancestral chloroplast variation.

In studies based on chloroplast markers, the interpretation of phylogenetic relationships within the genus *Prunus* is often considered in the context of subgeneric classification [1]. Similar clustering patterns have previously been noted in other phylogenetic studies of the genus. For example, Wan et al. (2023) showed that *P. cerasifera* and *P. domestica* clustered together with representatives of the subgenus *Prunus*, whereas *P. fruticosa* and *P. griffithii* var. *tianshanica* clustered within the subgenus *Cerasus* [32].

However, a strong geographical signal is observed in the dataset, as samples from Kazakhstan and Uzbekistan cluster together regardless of their species designation. This indicates that geographic origin is an important factor shaping chloroplast variation in the studied samples and that chloroplast haplotype distribution does not fully correspond to current taxonomic classification [4]. The observed clustering pattern may reflect shared historical chloroplast variation, regional demographic processes, or the limited resolution of chloroplast markers for distinguishing closely related *Prunus* taxa [33,34,35].

### 3.4. Potential Applications of the Developed Chloroplast Markers

In this study, three chloroplast regions (*matK_HpaII*, *ycf1_1* and *trnH–psbA*) showed potential for distinguishing the analyzed populations of *P. fruticosa* from the other studied taxa. Previously, in a study by Amar (2020) [36] the *ycf1* and *ndhF* regions were proposed as promising plastid markers for barcoding the genus *Prunus* at the cultivar level and for species identification. Furthermore, the chloroplast region *matK* is also widely used in plant DNA barcoding studies because of its ability to provide taxonomic information across different plant groups [37].

However, the selected chloroplast markers and the restriction endonucleases showed limited resolution for the other three species studied. This may be related to the recent divergence of these taxa but may also reflect the limited variability of the chloroplast regions analyzed. As a result, the analyzed chloroplast regions did not provide sufficient discriminatory power to distinguish these closely related taxa. Combining chloroplast and nuclear markers will likely provide a more complete picture of species boundaries and evolutionary relationships within *Prunus* [4].

## 4. Materials and Methods

### 4.1. Sampling

A total of 121 accessions of four wild cherry species—*Prunus erythrocarpa* (Nevski) Gilli (25), *P. verrucosa* Franch. (50), *P. griffithii* var. *tianshanica* (Pojark.) (32) Ingram and *P. fruticosa* Pall. (14)—were collected from natural populations located in different regions indigenous to Kazakhstan and Uzbekistan, as previously described in the study by Manapkanova et al. (2025) [13] (Table 5). The collection of plant material in protected areas of Kazakhstan and Uzbekistan was carried out with the permission of the relevant authorities.

Accessions of *P. griffithii* var. *tianshanica* (*n* = 32) were collected from four locations across two regions of Kazakhstan. Among these, 24 accessions originated from two isolated populations in the Almaty region (Kokpek Pass and Big Aksu).

Collection of *P. erythrocarpa* (*n* = 25) was conducted in two countries: in Kazakhstan, collections were made from two locations within a single region (*n* = 12), while in Uzbekistan, accessions were obtained from two regions, including two national park territories (*n* = 13).

*P. verrucosa* (*n* = 50) was collected in Kazakhstan from the same locations where *P. griffithii* var. *tianshanica* and *P. erythrocarpa* co-occur (*n* = 36). Additional sampling was carried out in Uzbekistan (*n* = 14), where this species also grows together with *P. erythrocarpa*.

*P. fruticosa* was collected only in the Kostanay region of Kazakhstan in three different locations (*n* = 14). In addition, 65 samples representing 17 wild *Prunus* species, with at least two accessions per species, from the Fruit Genebank of the Institute of Breeding Research on Fruit Crops, Julius Kühn Institute (JKI), Dresden-Pillnitz, were included in the analysis. The list of *Prunus* samples analyzed in this study is provided in Table S2.

Populations represented by a single individual were excluded from population-level diversity statistics (e.g., haplotype diversity and AMOVA) but were retained for haplotype distribution. For phylogeographic visualization, twenty-seven accessions labelled as *Prunus* spp. or *Prunus* hybr. and of unknown origin were excluded from the dataset (Table S2).

### 4.2. DNA Extraction and Development of Chloroplast Markers

Total DNA was extracted using the DNeasy^®^ Plant Mini Kit (QIAGEN, Hilden, Germany), which provides high DNA quality and stability required for the amplification of chloroplast loci. DNA was extracted from silica-dried leaf material. The quality of the extracted DNA was evaluated using Nanodrop (Thermo Scientific, Waltham, MA, USA).

*Chloroplast markers*. Six chloroplast primer pairs were developed based on selected sequencing data from different *Prunus* species (Table S3) located at the *matK*, *rpl16*, *ycf1* region 1 (*ycf1_1*), *ycf* region 2 (*ycf1_2*), *ndhF* and *trnH-psbA* chloroplast regions. Chloroplast genome sequences from representative *Prunus* species available in the NCBI database (accession numbers provided in Table S3) were used for comparative analysis. Sequence data for the respective chloroplast regions were retrieved from the NCBI database and aligned using the MEGA 12 software package [38]. Insertions/deletions (indels) and single nucleotide polymorphisms (SNPs) in the chloroplast genomes were identified using the alignment tool implemented in MEGA 12 [38] (Table 6). SNPs and indels were selected based on comparative alignments of chloroplast sequences from multiple Prunus species available in the NCBI database. Only variable sites showing consistent differences between the target taxa and other *Prunus* species were considered for marker development. In addition, selected SNPs were required to be suitable for CAPS analysis, including the presence of restriction enzyme recognition sites enabling CAPS-based discrimination among haplotypes and the possibility of designing specific primer pairs flanking the target regions for reliable PCR amplification.

Based on these results, specific primers were developed using the NCBI primer-designing tool (https://www.ncbi.nlm.nih.gov/tools/primer-blast/ accessed on 1 June 2026). In silico primer specificity was verified using NCBI Primer-BLAST version 2.5.0 by testing against available chloroplast and genomic sequences of *Prunus* species in the NCBI database. Primers targeting the *matK*, *ycf1* region 1 (*ycf1_1*), *ycf1* region 2 (*ycf1_2*), *rpl16*, and *trnH–psbA* regions were designed for fragment separation using a 3730XL DNA Analyzer (Applied Biosystems, Waltham, MA, USA). For this purpose, the forward primers were synthesized with an M13 adapter sequence according to Schuelke 2000 (M13: 5′CACGACGTTGTAAAACGAC3′) [39]. This adapter enables the incorporation of a fluorescent label during PCR, allowing visualization of PCR products on the 3730XL DNA Analyzer.

For the amplification of fragments of the *ndhF* chloroplast region, the primers were designed for separation on an agarose gel. This approach was used because multiple SNPs within the fragment were analyzed by restriction digestion, which would have removed the fluorescent label required for ABI-based fragment analysis. In addition, the fragment sizes were sufficiently large to be reliably detected by agarose gel electrophoresis.

### 4.3. Enzyme Restriction of PCR Products

To detect selected SNPs within the chloroplast regions *matK*, *ycf1* region 1, *ycf1* region 2, *rpl16*, *trnH–psbA*, and *ndhF*, CAPS analysis was performed using the restriction endonucleases *HpaII*, *ApoI*, and *HaeIII* (New England Biolabs, Frankfurt am Main, Germany) and *AluI* (Thermo Scientific, Darmstadt, Germany). PCR amplification was carried out using approximately 10 ng of template DNA. The resulting PCR products were subsequently digested with the respective restriction enzymes.

Restriction reactions were performed according to the manufacturers’ instructions using 0.7 U of enzyme per reaction in the appropriate buffer, followed by incubation at 37 °C for 2 h (*HpaII*, *ApoI*, *HaeIII*) or 12 h (*AluI*). The restriction reaction was heat-inactivated at 80 °C for 20 min.

Fragment profiles were visualized either by capillary electrophoresis using a 3730XL Genetic Analyzer (Thermo Scientific, Darmstadt, Germany) or by 2% agarose gel electrophoresis, depending on fragment size and resolution requirements. No direct sequencing of restriction fragments was performed; haplotypes were inferred from fragment length patterns consistent with in silico-predicted restriction profiles.

### 4.4. Haplotype Analysis

Haplotype analysis was performed on 121 accessions of four wild *Prunus* species (*P. erythrocarpa*, *P. verrucosa*, *P. griffithii* var. *tianshanica*, and *P. fruticosa*) using HAPLOTYPE ANALYSIS version 1.05 [40]. Haplotypes were defined by combining fragment size profiles across the analyzed loci, and each unique combination was assigned a haplotype number [40].

### 4.5. Phylogenetic Tree

A phylogenetic tree was constructed using the unweighted neighbor-joining method with 5000 bootstraps based on chloroplast markers for 121 wild *Prunus* accessions from Kazakhstan and Uzbekistan, together with the 38 accessions from the wild *Prunus* collection at the JKI in Dresden-Pillnitz, Germany, using DARwin ver.5 software [41]. The resulting tree was graphically refined, and clusters were annotated using Interactive Tree of Life (iTOL) software version 7.6 [42].

### 4.6. Analysis of Geographical Structure

To assess genetic relationships among species, principal coordinate analysis (PCoA), analysis of molecular variance (AMOVA), pairwise Φ*PT* values, pairwise population Nm (haploid) estimates derived from Φ*PT*, and Nei’s genetic identity were calculated using GenAlEx v. 6.5 [43,44]. Genetic distance matrices for multivariate analyses were calculated based on haploid chloroplast data using Nei’s genetic distance implemented in GenAlEx. PCoA was used to visualize genetic relationships among individuals and populations in a multivariate space. AMOVA was performed to partition genetic variation within and among populations and species. The significance of variance components was tested using 999 permutations. Pairwise Φ*PT* values were calculated based on haploid chloroplast variation as implemented in GenAlEx to estimate genetic differentiation among populations and species. Pairwise Nm values were derived from Φ*PT* as an indirect estimate of gene flow (reflecting seed-mediated inheritance of chloroplast DNA). A Mantel test was conducted to evaluate the correlation between genetic and geographic distances among populations using 999 permutations to test significance. Nei’s genetic identity was calculated based on haploid chloroplast data to assess genetic similarity among the four species. No missing data filtering was applied, as all loci were successfully amplified and scored across all samples.

## 5. Conclusions

Genetic differentiation between closely related taxa of the genus *Prunus* remains a complex and as yet unresolved issue. The present study was conducted for the first time using six chloroplast markers to investigate genetic variation in four wild *Prunus* species. The results highlight the limited chloroplast differentiation among three wild cherry species (*Prunus erythrocarpa*, *P. griffithii* var. *tianshanica*, and *P. verrucosa*) from Kazakhstan and Uzbekistan. The analyzed chloroplast markers revealed a strong association between chloroplast variation and geographic origin in the studied populations. However, the limited chloroplast resolution observed among Central Asian taxa indicates the need for further studies using nuclear markers and broader genomic approaches to better resolve species boundaries and clarify evolutionary relationships.

## Figures and Tables

**Table 1:** Haplotype diversity and related genetic diversity parameters across populations.

Species	Population	N	A	P	Ne	He
*Prunus erythrocarpa*	Kazakhstan, Turkestan region, Aksu-Zhabagly Nature Reserve	11	9	6	8.067	0.964
	Uzbekistan, Tashkent region, Ugam-Chatkal National Park	9	7	2	6.231	0.944
	Uzbekistan, Tashkent region, Ugam-Chatkal National Park	3	2	1	1.800	0.667
*Prunus fruticosa*	Kazakhstan, Kostanay region	7	5	1	4.455	0.905
	Kazakhstan, Kostanay region	3	2	0	1.800	0.667
	Kazakhstan, Kostanay region	4	2	0	1.600	0.500
*Prunus griffithii* var. *tianshanica*	Kazakhstan, Almaty region, Kokpek Pass	10	4	0	2.381	0.644
	Kazakhstan, Almaty region, Big Aksu	14	5	4	3.063	0.725
	Kazakhstan, Turkestan region, Sairam-Ugam National Park	4	4	2	4.000	1.000
	Kazakhstan, Turkestan region, Sairam-Ugam National Park	4	1	0	1.000	0.000
*Prunus verrucosa*	Kazakhstan, Turkestan region, Aksu-Zhabagly Nature Reserve	23	13	5	8.138	0.917
	Kazakhstan, Turkestan region, Sairam-Ugam National Park	5	3	0	2.273	0.700
	Kazakhstan, Turkestan region, Sairam-Ugam National Park	8	4	1	2.286	0.643
	Uzbekistan, Jizzakh region, Zaamin National Park	14	10	4	7.538	0.934
Mean		8.500	5.071	1.857	3.902	0.729

N = number of individuals; A = number of haplotypes; P = number of private haplotypes; Ne = effective number of haplotypes; He = gene diversity.

**Table 2:** Private chloroplast alleles in *Prunus* accessions in the gene bank of the Julius Kühn Institute (JKI) in Dresden-Pillnitz and their frequencies.

Species	Chloroplast Regions	Allele (bp)	Freq (>0.5)
*Prunus laurocerasus*	*matK*	366	1.0
*Prunus laurocerasus*	*matK_HpaII*	281	1.0
*Prunus laurocerasus*	*rpl16*	271	1.0
*Prunus maackii*	*rpl16*	259	1.0
*Prunus maximowiczii*	*trnH–psbA*	298	1.0
*Prunus padus*	*trnH–psbA*	305	1.0
*Prunus tomentosa*	*rpl16*	293	1.0
*Prunus fruticosa* Pall.	*ycf1_1*	269	0.95
*Prunus mahaleb*	*rpl16*	262	0.67
*Prunus mahaleb*	*trnH–psbA*	288	0.67

**Table 3:** Pairwise Population Matrix of Nei’s Genetic Identity based on 121 accessions of four wild *Prunus* species calculated from chloroplast CAPS markers.

*Prunus erythrocarpa*	*Prunus fruticosa*	*Prunus griffithii* var. *tianshanica*	*Prunus verrucosa*	
1.00				*Prunus erythrocarpa*
0.62	1.00			*Prunus fruticosa*
0.97	0.61	1.00		*Prunus griffithii* var. *tianshanica*
0.99	0.62	0.99	1.00	*Prunus verrucosa*

**Table 4:** Results of molecular variance analysis (AMOVA) among and within populations for each of the four studied *Prunus* species.

Source of Variation	df	SS	MS	Est. Var.	%	ϕ_PT_	*p* Value
*Prunus griffithii* var. *tianshanica*							
Among Pops	3	18.364	6.121	0.754	53%	0.534	0.001
Within Pops	28	18.386	0.657	0.657	47%		
*Prunus erythrocarpa*							
Among Pops	2	15.250	7.625	0.914	41%	0.411	0.001
Within Pops	20	26.141	1.307	1.307	59%		
*Prunus verrucosa*							
Among Pops	3	15.655	5.218	0.348	21%	0.210	0.001
Within Pops	46	60.185	1.308	1.308	79%		
*Prunus fruticosa*							
Among Pops	2	1.940	0.970	0.140	18%	0.182	0.001
Within Pops	11	5.417	0.492	0.411	82%		

df: degrees of freedom; SS: sum of squares; MS: mean sum of squares; Est. Var.: estimate of variance; ϕ_*PT*_: index of population genetic differentiation

**Table 5:** Collection of plant material in Kazakhstan and Uzbekistan.

Species	Population No	Collection Site	Latitude	Longitude	Number of Accessions
*Prunus griffithii* var. *tianshanica*	I	Kazakhstan, Almaty region, Kokpek Pass	43.4515	78.652667	10
	II	Kazakhstan, Almaty region, Big Aksu	43.35	79.611667	14
	III	Kazakhstan, Turkestan region, Sairam-Ugam National Park	43.0675	69.901833	4
	IV	Kazakhstan, Turkestan region, Sairam-Ugam National Park	42.953167	70.043	4
Total:					32
*Prunus erythrocarpa*	I	Kazakhstan, Turkestan region, Aksu-Zhabagly Nature Reserve	42.41375	70.4707	11
	II	Kazakhstan, Turkestan region, Sairam-Ugam National Park	41.52212	70.095	1
	III	Uzbekistan, Tashkent region, Ugam-Chatkal National Park	41.520183	70.023183	9
	IV	Uzbekistan, Tashkent region, Ugam-Chatkal National Park	41.15105	70.13955	3
	V	Uzbekistan, Jizzakh region, Zaamin National Park	39.6137167	68.430467	1
Total:					25
*Prunus verrucosa*	I	Kazakhstan, Turkestan region, Aksu-Zhabagly Nature Reserve	42.330917	70.37335	23
	II	Kazakhstan, Turkestan region, Sairam-Ugam National Park	42.667667	70.224333	5
	III	Kazakhstan, Turkestan region, Sairam-Ugam National Park	43.090833	69.924	8
	IV	Uzbekistan, Jizzakh region, Zaamin National Park	39.6084	68.445383	14
Total:					50
*Prunus fruticosa*	I	Kazakhstan, Kostanay region	53.170633	63.82645	7
	II	Kazakhstan, Kostanay region	53.288417	64.208833	3
	III	Kazakhstan, Kostanay region	52.438067	64.267233	4
Total:					14
Accessions total:					121

**Table 6:** Chloroplast DNA (cpDNA) used in this study.

Chloroplast Region	PCR Primer Forward	PCR Primer Reverse	Fragment Length Analysis	Expected Fragment Size (Not Restricted, bp)	Annealing Temp	Restriction Enzyme	Cutting Sequence	SNP-Sequence
*matK*	TTACGACTCTTTCTTCACGAG	GGGTATCCTTCGAAGCCAGA	3730XL DNA Analyzer	374	57 °C	*HpaII*	C ↓ CG ↑ G	ATCC[G/T]GTA
*ycf1_1*	ACAAAGTAAAATGTCTCTGTTCA	CGGTAGGGTCCATTCAAGAA		283	55 °C	*AluI*	AG ⇅ CT	GTTTA[G/T]CTTTTT
*ycf 1_2*	GTTAGAACTGTTCCGAATGA	TGGGTATTGGTATAAGCATCAGT		249	54 °C	*HaeIII*	GG ⇅ CC	GGC[C/T]TTTC
*trnH–psbA*	CCACTTGGCTACATCCGC	CGAGTTCTTGAAAGTAAAGGAGC		330	57 °C	N.A	N.A	N.A
*rpl16*	TGTTGAAAGAGTAAATATTCCCC	ACACGGTGAATAATATACGGA		251	54 °C	N.A	N.A	N.A
*ndhF*	CGTGTCGGGGATTTTGGTTT	AAAACAAGCAAGAGGTGGAAT	Agarose gel (2%)	696	57 °C	*AluI*	AG ⇅ CT	TTAATA[A/G]CTTGG
						*ApoI*	R **↓** AATT **↑** Y	AGCAGG[A/G]ATTTT
						*HpaII*	C **↓** CG **↑** G	GATC[C/T]GGAT

N.A: not applicable; **↓**, **↑**, ⇅: indicates the point of the cuts of the DNA strain by enzyme.

## Data Availability

The presented research results are included in the article and the Supplementary Materials. Further inquiries can be directed to the corresponding authors.

## Supplementary Materials

The following supporting information can be downloaded at: https://www.mdpi.com/article/10.3390/ijms27177566/s1.
